# Sensors at Centrosomes Reveal Determinants of Local Separase Activity

**DOI:** 10.1371/journal.pgen.1004672

**Published:** 2014-10-09

**Authors:** Fikret Gurkan Agircan, Elmar Schiebel

**Affiliations:** Zentrum für Molekulare Biologie der Universität Heidelberg, DKFZ-ZMBH Allianz, Heidelberg, Germany; Newcastle University, United Kingdom

## Abstract

Separase is best known for its function in sister chromatid separation at the metaphase-anaphase transition. It also has a role in centriole disengagement in late mitosis/G1. To gain insight into the activity of separase at centrosomes, we developed two separase activity sensors: mCherry-Scc1^(142-467)-ΔNLS^-eGFP-PACT and mCherry-kendrin^(2059-2398)^-eGFP-PACT. Both localize to the centrosomes and enabled us to monitor local separase activity at the centrosome in real time. Both centrosomal sensors were cleaved by separase before anaphase onset, earlier than the corresponding H2B-mCherry-Scc1^(142-467)^-eGFP sensor at chromosomes. This indicates that substrate cleavage by separase is not synchronous in the cells. Depletion of the proteins astrin or Aki1, which have been described as inhibitors of centrosomal separase, did not led to a significant activation of separase at centrosomes, emphasizing the importance of direct separase activity measurements at the centrosomes. Inhibition of polo-like kinase Plk1, on the other hand, decreased the separase activity towards the Scc1 but not the kendrin reporter. Together these findings indicate that Plk1 regulates separase activity at the level of substrate affinity at centrosomes and may explain in part the role of Plk1 in centriole disengagement.

## Author Summary

Centriole disengagement in telophase/G1 is the licensing step for centrosome duplication in the subsequent S phase. Recent data suggest that separase, together with polo-like kinase Plk1, is essential for the centriole disengagement and individual depletion of either separase or Plk1 alone fails to suppress the centriole disengagement. This raises the question of how separase activity is regulated at the centrosome. By generating a series of separase sensors, we show that separase at centrosomes becomes active already in mid metaphase, well before its activity can be detected at the chromosomes. Depletion of the previously published inhibitors of centrosomal separase, astrin or Aki1, did not promote separase activity at the centrosomes. This indicates that morphological criteria like the formation of multipolar spindles are insufficient criteria upon which to base predictions about separase regulation. Finally, the ability of Plk1 to promote cleavage of the Scc1-based reporter but not of the kendrin reporter reveals regulation of separase activity at the substrate level. These results provide partial explanation of the role of Plk1 in centriole disengagement.

## Introduction

Centrosomes are the main microtubule organizing centers of animal cells that consist of the organizing centrioles and pericentriolar material. Centrosomes, like DNA, duplicate exactly once per cell cycle. From S phase to the end of mitosis centrosomes are composed of a pair of centrioles, the mother and the daughter centrioles, which lie perpendicular to one another [1]. Separation of the mother and daughter centrioles, also referred to as “centriole disengagement”, takes place in telophase/G1 and is the licensing step for centriole duplication in the next S phase [2]–[4]. Following the centriole disengagement, a flexible linker containing the proteins C-Nap1 and rootletin assembles between the separated centrioles [5]. The C-Nap1/rootletin linker connects the two centrosomes (also named centrosome cohesion) until G2 or the beginning of mitosis when the linker is disassembled by the activity of the kinase Nek2 [6]–[9]. The disjoined centrosomes each containing two orthogonally engaged centrioles then become the poles of the mitotic spindle [9]. Thus, centriole engagement and centrosome cohesion are two distinct processes that are regulated by different mechanisms.

Separase (Espl1), a cysteine protease, is best known for its role in relieving sister chromatid cohesin during the metaphase-anaphase transition by cleaving the cohesin subunit Scc1/Rad21 [10], [11]. The function of separase in centriole disengagement has been established in *Xenopus* egg extracts [3]. Consistently, centriole disengagement was partially inhibited in human separase knockout cells. However, centriole disengagement was only blocked completely when the activities of both separase and the polo-like kinase Plk1 were simultaneously repressed [12].

Both cyclin B1 and securin have been shown to inhibit separase at chromosome until the end of anaphase [11], [13]. On the other hand, the regulation of separase at centrosomes is poorly understood. The proteins astrin and Aki1 have been proposed to act as inhibitors of centrosomal separase activity [14], [15]. Depletion of either astrin or Aki1 induces multipolar spindles in mitosis with disengaged centrioles, which would be consistent with premature separase activation [14], [15]. Furthermore, shugoshin (Sgo1) is the “guardian” of the chromosomes and prevents the prophase-dependent removal of cohesin from centromeres by recruiting PP2A-B56 to the centromere to counteract Plk1 kinase activity [16], [17]. Interestingly, a smaller version of Sgo1, called sSgo1, associates with the centrosomes. Depletion of Sgo1 promotes centriole disengagement in human cells in a manner that requires Plk1 activity [18].

Kendrin, a splice variant of pericentrin, is a coiled-coil motif containing protein which localizes to the centrosomes, where it recruits the γ-tubulin ring complex and modulates centrosome cohesion through the regulation of Nek2A kinase activity [19]–[21]. Localization studies identified also the subunits of cohesin at the centrosomes [22]. Moreover, siRNA depletion experiments showed that not only kendrin, but also cohesin is important for the integrity of the centrosome [23]. Strikingly, the cohesin subunit Scc1/Rad21 and kendrin/pericentrin B, here referred to as Scc1 and kendrin, respectively, are both cleaved by separase at the centrosome [24], [25]. Subsequent biochemical analyses support the notion that cohesin is the “glue” that connects the mother to the daughter centrioles, and that cleavage of this pool of cohesin by separase promotes centriole disengagement [24]. Furthermore, expression of a non-cleavable version of kendrin blocks centriole disengagement [25].

A fluorescence-based method was used to measure the separase activity on chromosomes [26], [27]. This separase activity sensor comprises the separase cleavage sites of Scc1^142-467^ flanked by N-terminal mCherry and C-terminal eGFP fluorescent molecules. Cleavage of the sensor releases the eGFP moiety to diffuse throughout the cytoplasm while the mCherry remains anchored at chromosomes because it is fused to C-terminus of H2B. As a result, the color of the sensor at chromosomes switches from yellow to red [26].

Here we investigated the regulation of separase activity at the centrosomes using Scc1- and kendrin-based separase sensors, which were targeted to centrosomes via the PACT domain of AKAP450 [28], [29]. Both sensors changed their fluorescent signal in a manner that required separase activation and the integrity of the separase cleavage site. Centrosomal separase activity strongly increased midway through metaphase ahead of chromatin-associated separase activity. We also tested whether astrin, Aki1 and sSgo1 regulate centrosomal separase activity and show that morphological criteria are insufficient indicators for separase activation at centrosomes.

## Results and Discussion

### Detection of Separase Activity at the Centrosome Using Separase Sensor

**Figure 1:**
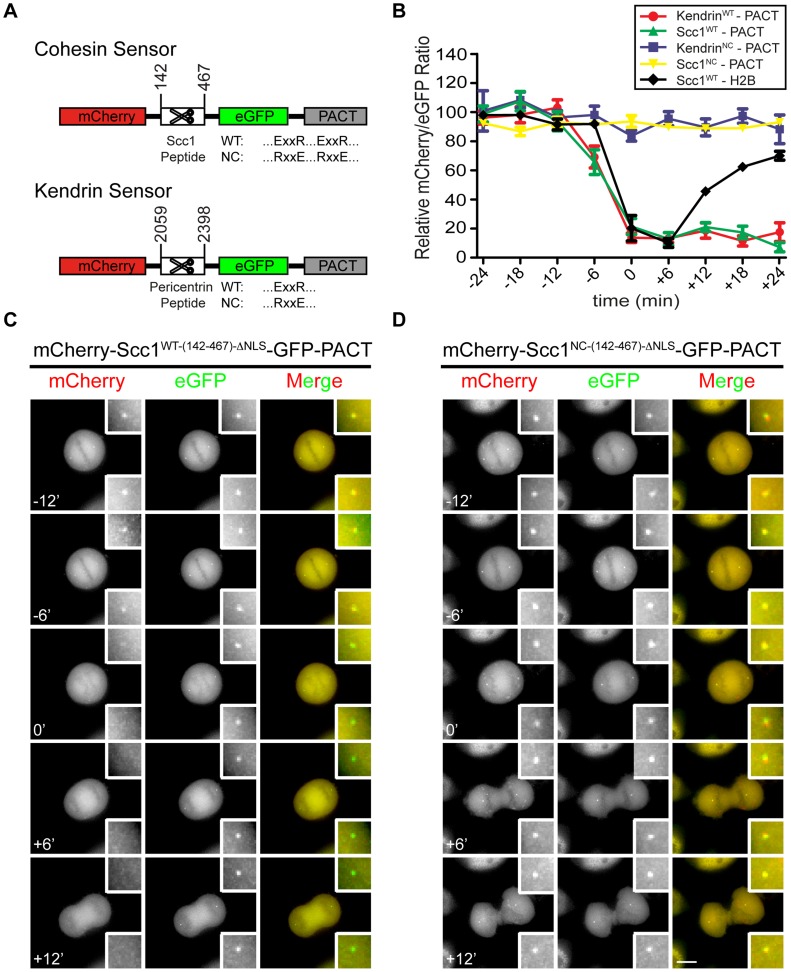
Construction of separase sensor constructs for centrosomes. (A) The architecture of the centrosomal Scc1 and kendrin sensors. (B) Relative intensity plots of the mCherry/eGFP ratio for the following centrosomal signals: mCherry-Scc1^WT-(142-467)-ΔNLS^-eGFP-PACT (n = 18), mCherry-Scc1^NC-(142-467)-ΔNLS^-eGFP-PACT (n = 20), mCherry-Kendrin^WT-(2059-2398)^-eGFP-PACT (n = 27), and mCherry-Kendrin^NC-(2059-2398)^-eGFP-PACT (n = 11). In addition, H2B-mCherry-Scc1^(142-467)^-eGFP (n = 10) at chromatin was analyzed for separase activity; for H2B sensor, eGFP/mCherry ratio was used and plotted as described in materials and methods section. The ratio of mCherry/eGFP was normalized to the average of the first two data points (time = −24 min and −18 min) in order to assess cleavage rates. Time t = 0 indicates anaphase onset. The error bars are SEM. (C, D) The mCherry-Scc1^WT-(142-467)-ΔNLS^-eGFP-PACT (C) and mCherry-Scc1^NC-(142-467)-ΔNLS^-eGFP-PACT (D) sensors stably integrated into the FRT locus of HeLa T-REx cells were followed every 6 min. Time t = 0 min indicates anaphase onset. Representative images are shown. Insets show the two-fold enlarged centrosomal signals. Scale bar: 10 µm.

Separase localizes to centrosomes during mitosis where it regulates the centriole disengagement [12], [30]. However, it remains to be established how this centrosomal separase activity is regulated. To address this important question, we have developed two distinct “separase sensors” that measure separase activity at the centrosomes of individual cells in real time. The sensors contained the separase cleavage sites (SCS) of either Scc1 (142–467 aa; cleavage sites at R^172^ - cleavage site 1, R^450^ and R^460^ - cleavage site 2) or kendrin (2059–2398 aa; cleavage site at R^2231^), the two known centrosomal separase substrates [26], [31]. mCherry was fused to the N-terminus and eGFP to the C-terminus of each SCS element. Each mCherry-SCS-eGFP module was joined to the N-terminus of the PACT domain of AKAP450 (aa 3643–3808) (Figure 1A, Figure S1A). The PACT domain is a high affinity centrosomal targeting domain, and so will target each of these two reporters to the centrosome [28]. To control for separase cleavage dependent changes in fluorescent signal, we also used reporters in which critical residues within the separase cleavage sites (ExxR) were mutated (RxxE). These mutations inhibit the ability of separase to cleave the fusion protein (separase sensor^NC^) (Figure S1A) and so this modified reporter serves as an important internal control for separase dependent cleavage [10], [26].

The reporters were stably integrated into the FRT sites of HeLa T-REx cells to render their expression dependent upon the addition of doxycycline [32]. The first version of the Scc1-derived sensor accumulated in the nucleus during interphase and failed to bind to the centrosomes even after nuclear envelope breakdown in mitosis (Figure S1B. However, inactivation of the first nuclear localization sequence (ΔNLS-1, 319–323 aa of Scc1) in the mCherry-Scc1^(142-467)-ΔNLS^-eGFP-PACT reporter (named Scc1^(142-467)-ΔNLS)^) promoted its binding to the centrosomes (Figure S1C). Nonetheless a fraction of the sensor was still detected in the cytoplasm and nucleus. Most likely the number of centrosomal binding sites for the PACT based reporter is limited as this has been observed for other PACT fusion proteins [29]. Fluorescence recovery after photobleaching (FRAP) revealed that the mCherry-Scc1^(142-467)-ΔNLS^-eGFP-PACT reporter stably associated with centrosomes (Figure S1D). The kendrin-based sensor was also enriched at the centrosome (Figure S1C, right panel) with additional signal in the cytoplasm. Thus, both reporters are targeted to centrosomes via their PACT domain.

At chromosomes, separase becomes active just before anaphase onset [26]. In contrast, the exact timing of separase activation at centrosomes is unknown. Real time fluorescence analysis showed that the yellow mCherry-SCS-GFP-PACT signal at centrosomes switched to green GFP-PACT before cells entered anaphase (Figures 1B, C and Figure S1E). This was indicative of reporter cleavage to release the mCherry moiety into the cytoplasm, while eGFP-PACT was retained at centrosomes. The yellow fluorescent signal from the corresponding non-cleavable separase sensor^NC^ persisted at centrosomes throughout the cell cycle (Figures 1B, D and Figures S1E, F). It has been shown that both Scc1 and kendrin are substrates of separase, and they are not cleaved upon Espl1 siRNA [10], [25]–[27], [33], [34]. For proof of principle analyses, we interfered with separase activity via Espl1 siRNA and showed that reporter activation was prevented even when cells passed into G1 phase (Figures S2A–E). Thus, the cleavage of the separase reporters at the centrosome was indeed dependent on separase activity and occurred before anaphase onset.

We next addressed whether the timing of separase activation on chromosomes coincided with its activation on centrosomes. For this, we compared cleavage of the centrosomal mCherry-Scc1^(142-467)-ΔNLS^-eGFP-PACT and mCherry-Kendrin^(2059-2398)^-eGFP-PACT reporters with the chromatin-associated sensor. Anaphase onset was used as the internal reference point (t = 0) for both reporters. In line with published data, separase became active at chromosomes between −6 and 0 min before anaphase onset [26]. Both centrosomal reporters, however, were activated between −12 and −6 min, clearly before separase activation on chromatin (Figure 1B, Figure S3). The delayed activation of separase at chromatin might reflect a preferred spatial activation of separase at centrosomes, as has been reported for Cdk1-cyclin B1 [35]. The degradation of cyclin B1 in *Drosophila* also starts at spindle poles and from there spreads to the metaphase plate [36]. This behavior of the separase inhibitor cyclin B is consistent with the earlier activation of separase at centrosomes. In such a model, separase activity spreads from centrosomes to the cytoplasm. However, it is also possible that separase is activated with the same timing at both locations but cleaves cohesin and kendrin at the centrosomes faster than the chromatin bound cohesin because of topological restrains.

### Recruitment and Activation of Separase at Centrosomes Are Two Distinct Steps That Do Not Require Microtubules

**Figure 2:**
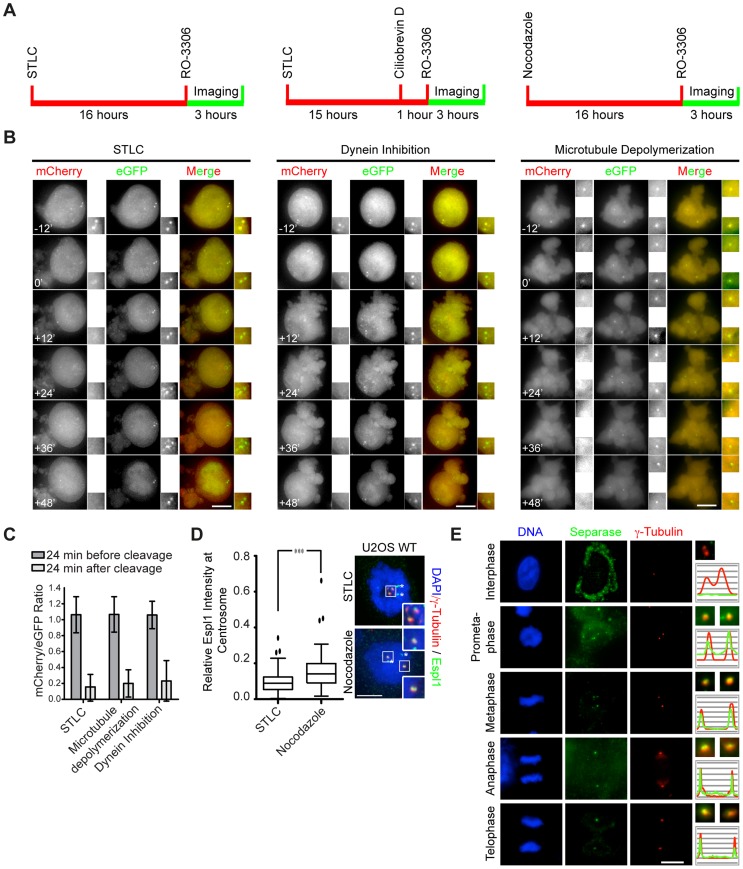
Separase localizes to centrosomes in a microtubule-independent manner. (A) Cells were arrested at prometaphase with either 5 µM of STLC or 0.1 µg/ml of nocodazole. To inhibit dynein, STLC arrested cells (15 h) were treated for 1 h with 50 µM of cytoplasmic dynein inhibitor Ciliobrevin D. All cells were then treated with 4 µM of the Cdk1 inhibitor RO-3306 to force mitotic exit. Cells were imaged every 6 min for sensor cleavage. (B) Representative images showing separase activity in the presence of the Eg5 inhibitor STLC, dynein inhibitor Ciliobrevin D or the microtubule poison nocodazole. Time t = 0 min reflects initiation of mitotic exit, which was judged upon the cleavage of the sensor and a sudden shift in focus that arises from cell spreading at the end of mitosis. Insets show the two-fold enlarged centrosomal images. Scale bar: 10 µm. (C) The mCherry/eGFP ratio of (B) as measured 24 min before and after cleavage (t = 0 min), and normalized to the initial ratio (t = −24 min). Error bars represent S.D. n = 20. (D) Quantification of separase signal at the centrosome after normalization to the centrosomal γ–tubulin signal. The relative Espl1 intensity was plotted using Graphpad 6 with Whisker-Box Plot. n = 98 (nocodazole), n = 70 (STLC) (error bars = SD, *** represents p<0.001). Separase signal at centrosomes does not decrease upon depolymerization of microtubules. The asterisks show unavoidable antibody background. Insets show the two-fold enlarged images of the boxed area in the main image. (E) Localization of separase at centrosomes during mitosis. Indirect immunofluorescence with anti-separase antibody was performed. γ-tubulin was used as centrosomal marker. Two-fold enlargements of the centrosomes are depicted on the right. The distribution of the green separase and the red γ-tubulin signals are presented as line scans. Scale bar: 10 µm.

A number of proteins are transported to the centrosomes along microtubules [37], [38]. We used the Scc1-based reporter to ask whether activation of separase at the centrosomes requires microtubules or the activity of the minus-end directed, microtubule-based motor protein dynein. Cells were first synchronized in prometaphase with the drugs STLC ((+)-S-trityl-L-cysteine) or nocodazole that arrest the cells due to spindle checkpoint activation (Figure 2A) [39]. In STLC-treated cells, dynein was subsequently inhibited by the Ciliobrevin D [40]. Cells were then driven from prometaphase to G1 by Cdk1 inhibition with RO-3306, and as previously reported this treatment does not interfere with the activation of separase during mitotic exit (Figure 2A) [41], [42]. Moreover, in vivo experiments revealed that centrioles still disengage in response to Cdk1 inhibition [12].

Upon dynein inhibition or microtubule depolymerization the Scc1-based reporter was cleaved with similar efficiency as in the control (Figure 2B, C) or during a normal mitotic exit (Figure 1B, 24 min). Thus, separase activity at centrosomes is independent of polymerized microtubules or dynein activity. Consistently, the level of Espl1 at the centrosome did not decrease by nocodazole-induced microtubule depolymerization when compared with STLC treated control cells (Figure 2D). Instead, nocodazole slightly increased the centrosomal Espl1 signal (Figure 2D).

Scc1/Rad21 is also a substrate of caspase-3 during apoptosis [43]. We analyzed the level of PARP cleavage in order to eliminate the involvement of caspase-3 in sensor cleavage in our experimental approaches (Figure S4A). Although there was no significant change in apoptotic cleavage of PARP, in order to be completely sure that the apoptotic cleavage of Scc1-based sensors did not interfere with the experimental setup, the experiments were repeated with either a non-cleavable version of the separase sensor (Figure S4B) or with the wild-type sensor in the presence of the apoptosis inhibitor Z-VAD-FMK (Figure S4C). As expected, the non-cleavable sensor was not subject to cleavage and the wild-type sensor was still cleaved in the presence of apoptosis inhibitor. In conclusion, centrosomal activation of separase is independent of microtubule integrity and reporter cleavage is a direct consequence of separase activity at centrosomes.

Activation of separase midway through metaphase prompted us to analyze the localization of separase to discriminate between two possibilities: First, separase is targeted to the centrosomes after initial activation at the centrosome. Alternatively, binding to the centrosome and local activation are two distinct steps. Although we detected a cytoplasmic separase signal during interphase as previously reported [44], no centrosomal signal was seen. From pro-metaphase onwards until telophase/G1 separase was detected at centrosomes (Figure 2E). Furthermore the localization of separase-GFP, expressed in HeLa BAC cells, confirmed the timing of separase recruitment to centrosomes (Figure S5). Thus, separase localizes to centrosomes from prometaphase to G1 but is only active at this location 6–12 min before anaphase onset. This means that separase targeting to centrosomes and separase activation are two independent processes.

### Regulation of Separase Activity at Centrosomes

**Figure 3:**
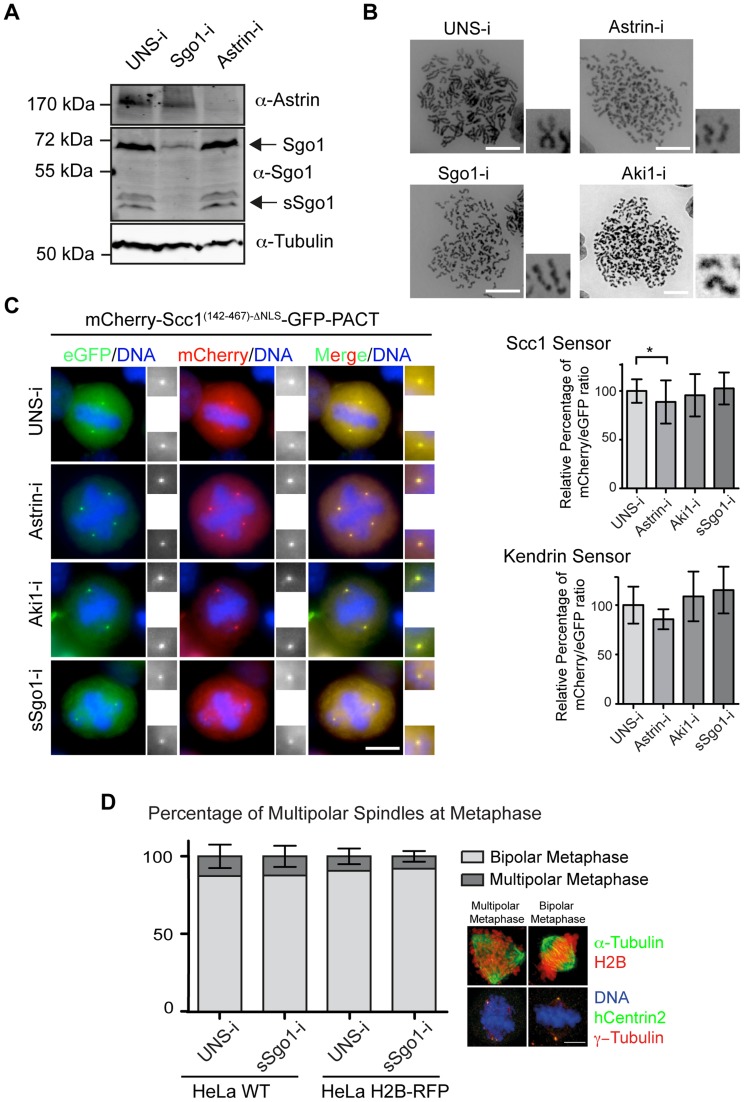
Astrin (Spag5), Aki1 and Sgo1 depletion does not promote centriole disengagement. (A) Immunoblot analysis of HeLa cells following astrin and sSgo1/Sgo1 depletion. α-Tubulin was used as loading control. (B) Depletions of Sgo1, astrin or Aki1 caused premature loss of sister chromatid cohesin. Chromosome spreads of HeLa cells are shown. Four fold enlargements on the right show the separated sister chromatids. Scale bar: 10 µm. (C) Representative images of HeLa cells stably expressing the Scc1 and kendrin sensors upon depletion of Sgo1, astrin or Aki1. The two-fold enlargements show the centrosomes. The relative mCherry/eGFP ratios upon siRNA treatment of cells are depicted on the right for the Scc1 (n>45) and kendrin (n>15) sensors. One-way ANOVA was used as statistical test (* represents p<0.05). Error bars are SD. Scale bar: 10 µm. (D) The percentages of multipolar spindles did not change in response to Sgo1 depletion in HeLa WT and HeLa H2B-RFP cells. The images on the right indicate examples of cells with multipolar and bipolar metaphase spindles. Scale bar: 10 µm. n = 30. Error bars indicate SD.

The proteins astrin (Spag5) and Aki1 have been implicated in the regulation of separase activity at centrosomes [14], [15]. Depletion of astrin causes premature separase activation and sister chromatid disjunction [14]. Moreover, depletion of either astrin or Aki1 promotes premature centriole disengagement in mitosis [14], [15]. To test whether astrin and Aki1 directly regulate separase activity at centrosomes, each protein was depleted from HeLa cells carrying either the Scc1- or the kendrin-based separase sensors by siRNA (Figure 3A, Figure S6A). Consistent with published data, astrin or Aki1 depletion gave rise to cells with separated sister chromatids (Figure 3B) [14]. However, the Scc1 and kendrin sensors indicated that separase was either not or only very weakly activated at centrosomes (Figure 3C). Furthermore, there was no increase in the number of separated centriole pairs upon astrin depletion since always a mother and daughter centriole pair closely associated with one mitotic spindle pole (Figures S6B, C). It is therefore unlikely that astrin regulates centrosomal separase activity. Similarly, Aki1 depletion did not activate separase (Figure 3C).

Disengaged centrioles remain connected by centrosomal linker proteins including rootletin until onset of mitosis [4]. To eliminate the possibility that in our depletion experiments mitotic centrioles are joined together by the C-Nap1/rootletin linker, we asked whether the protein rootletin was associated with centrosomes [45]. Rootletin was absent from the mitotic centrosomes upon astrin and Aki1 depletion while it was associated with interphase centrioles (Figure S6B). This provides further evidence that mitotic centrioles remain together when astrin and Aki1 are depleted.

Thus, how does astrin or Aki1 depletion cause sister chromatid disjunction without separase activation? Recent reports have implicated the role of astrin in kinetochore-microtubule attachments [46]. Aki1 or astrin depletion probably causes mitotic arrest through activation of the spindle assembly checkpoint that eventually leads to loss of sister chromatid cohesion without separase activation through a mechanism called “cohesion fatigue” [47].

A smaller splice variant of Sgo1, named sSgo1, associates with centrosomes [18]. It has been reported that Sgo1/sSgo1 depletion leads to both the premature disjunction of sister chromatids and centriole disengagement. sSgo1 might counteract Plk1 activity through the recruitment of PP2A-B56, as demonstrated for centromeric Sgo1 [16], [48], [49]. Alternatively, sSgo1 may regulate centrosomal separase activity. To discriminate between these two possibilities, Sgo1 and sSgo1 were co-depleted from our reporter cell line by siRNA (Figure 3A). Depletion of sSgo1 co-depletes Sgo1 because the coding region of this splice variant overlaps with Sgo1. Chromosome spreads revealed that Sgo1/sSgo1 depletion arrested mitotic progression at metaphase with disjoined sister chromatids (Figure 3B). However, Sgo1/sSgo1 depleted cells did not separate the closely associated centrioles prematurely as judged by the persistence of paired centrin signals (Figures S6B, C). Measurements of the distance of the distal centriole marker GFP-centrin also confirmed the tightly association of mother and daughter centrioles in Sgo1/sSgo1 depleted cells (Figure S6D). The centriole pairs in siRNA Sgo1/sSgo1 depleted cells had the same close distance as wild type cells arrested at prometaphase by STLC. In contrast, STLC treated cells that were driven into G1 by Cdk1 inhibition and therefore had disengaged centrioles showed much larger GFP-centrin distances (Figure S6D) [3]. Moreover, we failed to see a significant increase in the percentage of multipolar spindles during mitosis, which is normally caused by premature centriole disengagement (Figure 3D) [50].

Sgo1/sSgo1 depletion did not activate separase at centrosomes or chromosomes as indicated by the co-localization of eGFP and mCherry at centrosomes and chromosomes (Figure 3C, Figure S6E). However, in ∼20% of Sgo1/sSgo1 siRNA cells, we observed unfocused γ-tubulin signals at centrosomes that sometimes contained extra γ-tubulin foci (Figure S6F). The majority of these additional γ-tubulin signals did not contain GFP-centrin suggesting that they arose from disruption of centrosome structure rather than from centriole disengagement. Indeed, loss of tension at kinetochores provokes centrosome fragmentation [51]. Thus, Sgo1/sSgo1 depleted cells fragment the centrosomes due to the loss of kinetochore tension.

### Plk1 Is Important for Cleavage of the Scc1 but Not the Kendrin Sensor

**Figure 4:**
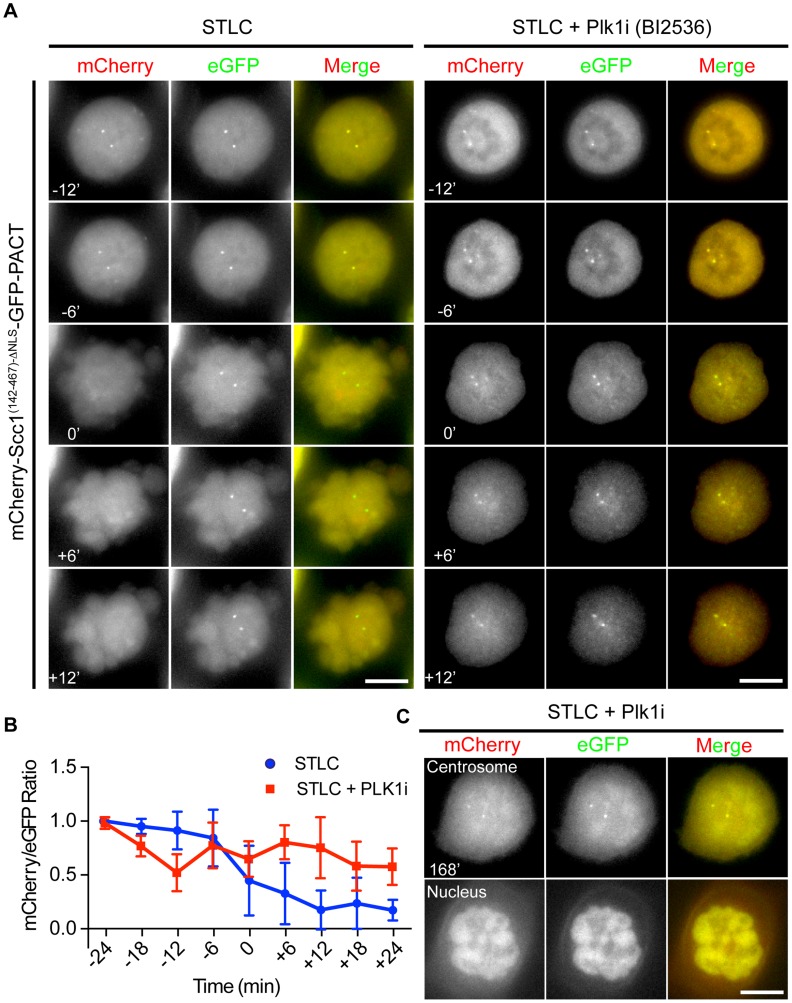
Plk1 is important for cleavage of the Scc1 sensor. (A) HeLa cells were incubated with 5 µM STLC and with or without 100 nM BI2536 for 1 h. Cells were driven out of mitosis with 4 µM of Cdk1 inhibitor RO-3306 in order to observe the activity of separase. Single Z-plane images were depicted. Size bars: 10 µm. (B) Quantification of (A). Separase activity decreases by 50% upon Plk1 inhibition although the cells exited mitosis. n≥10. Error bars represent SD. (C) Chromosome condensation of cells from (A) after 168 min incubation with Cdk1 inhibitor. Upper panel shows the Z-plane where the centrosomes were in focus, and the lower plane shows the Z-plane where the nucleus was in focus. Note that a fraction of the mCherry-Scc1^(142-467)-ΔNLS^-GFP-PACT sensor accumulates inside the nucleus. Scale bar: 10 µm.

Plk1 has been suggested to promote centriole disengagement in a pathway overlapping with separase [12]. This function of Plk1 directed us to test whether Plk1 regulates centrosomal separase activity. We first arrested cells with the kinesin-5 (Eg5) inhibitor STLC in prometaphase. Subsequently, Plk1 activity was inhibited using the specific, small molecule inhibitor BI2536. Cdk1 inhibition (RO-3306) then drove cells out of mitosis (Figure S7A). Plk1 inhibition reduced cleavage of the Scc1 based separase sensor at centrosomes to ∼50% even when cells were incubated for 3 h with the Cdk1 inhibitor (Figure 4A, B). After Cdk1 inhibition the nuclear envelope reformed in 100% of the cells and a fraction of the Scc1 sensor was targeted to the nucleus due to its nuclear localization signal indicating that cells exited mitosis (Figure 4C and Figure S1C). Moreover, inhibition of Plk1 decreased the activity of separase not only at the centrosome but also at the chromatin (Figure S7B).

**Figure 5:**
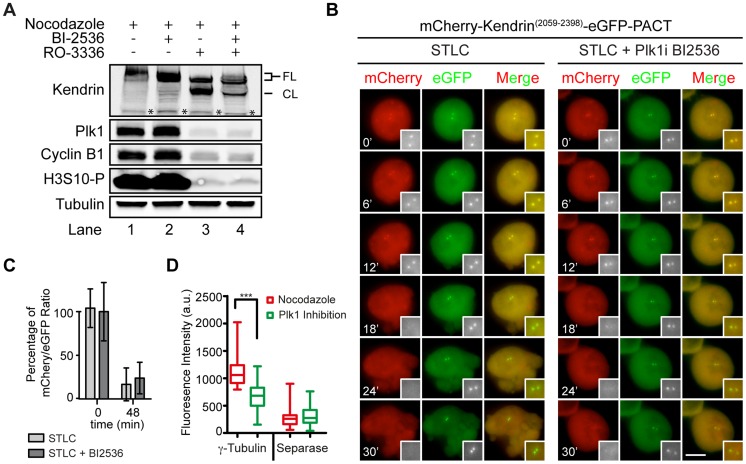
Plk1 regulates substrate cleavage of Scc1 but neither the activity of separase nor the cleavage of kendrin. (A) The level of cyclin B1 and cleaved kendrin do not change upon Plk1 inhibition. FL indicates the non-cleaved kendrin and CL the cleaved kendrin product. The asterisk indicates protein bands that are unspecifically recognized by the anti-kendrin antibody. (B) mCherry-Kendrin^(2059-2398)^-eGFP-PACT stably expressing HeLa cells were analyzed for the cleavage of the sensor upon Plk1 inhibition. Experiment was performed as described in Figure 4A. The two-fold magnifications on the bottom right of the figures highlights the centrosomes. The scale bar represents 10 µm. (C) The quantification of (B). mCherry/eGFP ratio at centrosomes was quantified just after (t = 0) and 48 min after (t = 48) addition of the Plk1 inhibitor BI2536. Data were normalized to the average mCherry to eGFP ratio at t = 0 min. (D) The levels of γ-tubulin and Espl1 signals at the centrosome were plotted using Whisker-Box plot after Plk1 (BI2536) inhibition. The level of Espl1 did not change upon Plk1 inhibition although the γ-tubulin signal declined compared to nocodazole treatment as a control to maintain the cells at prometaphase. Statistical analysis was performed with one-way ANOVA (*** represents p<0.001). N≥30, Error bars: SD.

The impact of Plk1 towards the activity of separase at the centrosome could be due to the decrease in the phosphorylation of the anaphase promoting complex APC/C. Plk1 phosphorylation of APC/C complex in G2 cells maintains the APC/C in an inactive state, such that inhibition of Plk1 induces premature APC/C activation in G2 [52]. In order to test, whether we see a similar effect during mitosis, we analyzed the steady state levels of the APC/C substrate cyclin B1. Cyclin B1 levels of prometaphase arrested cells (nocodazole) did not increase in repeated experiments following Plk1 inhibition (Figure 5A, lines 1 and 2). However, kendrin was still efficiently cleaved when cells were driven out of mitosis by the Cdk1 inhibitor RO-3336 as indicated by Plk1/Cyclin B1 degradation and H3S10 dephosphorylation (Figure 5A, lanes 3 and 4). These findings argue against the possibility that Plk1 inhibition during mitosis activates the APC/C that then would degrade the separase inhibitors securin and cyclin B1 to promote separase activation.

Alternatively, Plk1 phosphorylation of Scc1 might increase its cleavage. Such a model has been proposed for the chromosomal Scc1 [49]. Scc1 has two separase cleavage sites (R172 and R450/R460) with neighboring Plk1 phosphorylation sites (e.g. S175 and S454). At chromosomes Plk1 mainly regulates the Scc1 cleavage site at R450/R460. Cleavage at R172, moreover, is only moderately stimulated by Plk1 [49]. To test whether Plk1 stimulates Scc1 cleavage at centrosomes through substrate phosphorylation, we first inactivated the separase cleavage at R172 (mutation from “ExxR” to “RxxE”). Additionally, we mutated the Plk1 phosphorylation site Ser454 to Ala to prevent phosphorylation near the second separase cleavage site. Inactivation of the first separase cleavage site by the R172E mutation strongly reduced cleavage of the Scc1 reporter indicating that the first separase cleavage site around R172 is preferentially cleaved over the second site at R450/R460 (Figure S8A, B).

We next asked whether the R450/R460 site is regulated through Plk1 phosphorylation at S454. The R172E/S454A double mutation completely abolished separase cleavage of the Scc1 reporter (Figure S8A, C). Cleavage of the first site at R172 was independent of the Plk1 phosphorylation site S175 as the double phospho-dead mutant (S175A/S454A) was still cleaved by separase (Figures S8A, D) as reported before [49]. However, Plk1 inhibition by BI2536 in S175A/S454A mutant cells prevented the complete cleavage of this mutant sensor (Figure S8E). This implies that additional Plk1 sites in Scc1 might be important for the separase activity at the centrosome. Additional Plk1 phosphorylation sites close to the fist separase cleavage site of Scc1 have been reported [49]. Mutating these sites (T144A, S153A, S175A, S185A, T186A, T187A, T188A, S189A) together with inactivating the second separase cut site (R450E and R460E, Scc1^(142-467)-8A-ΔNLS-ERRE^) strongly reduced cleavage efficiency of the Scc1 separase reported at centrosomes (Figures S9A, B). Thus, separase cleavage of both cut sites of Scc1 at centrosomes is promoted by Plk1 phosphorylation.

Interestingly, the same Plk1 inhibition experiment with the kendrin sensor did not reveal a dependency of sensor cleavage on Plk1 activity (Figures 5B and C). This result excludes a role for Plk1 in separase targeting to the centrosomes or in separase specific activity since both reporters would be equally affected if this were the case. Consistently, separase's localization on centrosomes was not affected by Plk1 inhibition when compared with nocodazole arrested prometaphase cells (Figure 5D). In contrast, Plk1 inhibition affected localization of γ-tubulin at centrosomes implying that Plk1 inhibition worked as expected (Figure 5D) [53]. Taken together, a likely explanation of our results is therefore that Plk1 activates the Scc1 substrate whereas such an activation step is not required for kendrin.

In conclusion, we have constructed reporter proteins that measure the activity of separase at centrosomes. With these sensors in hand, we have tested putative regulators of centrosomal separase. This analysis indicates that astrin and Aki1 do not activate separase at centrosomes. Instead, we propose that the centriole separation phenotype that arises from astrin and Aki1 depletion is a secondary consequence of the loss of sister-chromatid cohesion [14], [18]. This demonstrates the importance of using separase sensors to analyze separase activity at centrosomes. Morphological criteria such as multipolar spindles or multiple centrosomes do not support conclusions about separase activity at centrosomes as these phenotypes may arise from the loss of sister chromatid cohesion during prolonged mitotic arrests. The sensors we have constructed are excellent tools to find the regulators of separase at the centrosome in screening-based studies.

Our data suggest that separase localizes to centrosomes from prometaphase until the end of mitosis with continued accumulation to enhanced levels during anaphase. Separase was active at centrosomes before it was activated at chromosomes, which may be explained by the early loss of Cdk1-cyclin B1 activity at the centrosomes ahead of the general wave of cyclin B1 degradation [36], [54], [55]. The APC/C complex may be initially activated at centrosomes before diffusing throughout the cell. We also found that Plk1 promotes cleavage of a subset of substrates by separase at centrosomes, while kendrin does not require Plk1 activation for separase cleavage. This finding at least in part explains the role of Plk1 in centriole disengagement.

## Materials and Methods

### Antibodies

The following antibodies directed against the indicated proteins were used in this study: Sgo1 (1∶1000, Thermo Scientific PA5-30869), astrin (1∶1000, Bethyl Laboratories A301-512A), tubulin (1∶1000, Sigma T9026), pericentrin (1∶2000, Abcam ab4448), cyclin B1 (1∶200, CR UK V152), PARP (1∶1000, Cell Signaling #9532), separase (1∶500, Abcam ab16170) for WB, separase (1∶500, Abcam ab3762) for IF and γ-tubulin (1∶1000, Sigma).

### Cell Culture

HeLa Centrin2-GFP cells, HeLa FRT cells, Separase-GFP expressing HeLa BAC cell line and U2OS cells were cultured in Dulbecco's Modified Eagle's Medium (DMEM) Glutamax (Gibco) supplemented with 10% FBS, 1% P/S and 1% Na-Pyruvate. Stable HeLa FRT cells were created and sustained as described previously [56].

At least two different siRNAs were used for depletion experiments of astrin, Aki1 and sSgo1. siRNA oligos that were directed against the same mRNA gave identical phenotypes in depletion experiments. Therefore, the results of only one siRNA oligo (marked with *) per mRNA are shown in this manuscript. Cells were transfected with cDNA or siRNA according to the manufacturer's protocol via Lipofectamine 2000 or RNAiMax, respectively (Invitrogen). Sgo1 siRNAs (s45599*: 5′-CAUCUUAGCCUGAAGGAUAtt-3′ and s45600: 5′-GGCAAACGCAGGUCUUUUAtt-3′, Ambion; #L-015475-00-0005 pool of: 5′-GUGAAGGAUUUACCGCAAA-3′, 5′-AAACGCAGGUCUUUUAUAG-3′, 5′-GUUACUAUCUCACAUGUCA-3′, 5′-CAGCCAGCGUGAACUAUAA-3′, Dharmacon) were used at concentration of 100 µM. Aki1 siRNA (#s226792*: 5′-AACAAAGACAUCCAGAUCGCCAGGG-3′, Ambion; #GS54862 pool of: 5′-CACGAGCGCATCGTCAAGCAA-3′, 5′-CAGCGCCAAGATGCGGCGCTA-3′, 5′-CAAGTTCGAAGTGGTTCACAA-3′ and 5′-CCCGGCGTCCACGCCTACCTA-3′, Qiagen), astrin siRNA (#SI02653938: 5′-AAAUUAGCUCUACUCCUAAtt-3′ and #SI02653945*: 5′-CCGACAACUCACAGAGAAAtt-3′), Espl1 siRNA (#s121651: 5′-GCUUGUGAUGCCAUCCUGAtt-3′, Ambion) were used at concentration of 50 µM. Sgo1 depleted cells were checked after 24 h whereas astrin, Aki1 and Espl1 depleted cells were checked 48 h after transfection via microscopy and immunoblotting.

### Cell Synchronization and Treatment

For live cell imaging, cell cycle progression was blocked with 2.5 mM thymidine (Sigma, #T1895) for 24 h. After three washes with PBS, cells were released into fresh media for 10 h. For inhibitor experiments, cells were arrested in prometaphase with 5 µM S-trityl-L-cysteine (STLC, Sigma #164739) for 15 h, and Plk1 inhibitor BI2536 or 50 µM dynein inhibitor Ciliobrevin D (#250401, Millipore) was added for 1 h more. 5 µM of Cdk1 inhibitor RO-3336 (Millipore, #217699) was used to trigger mitotic exit. Z-VAD (OMe)-FMK was used for apoptosis inhibition (Millipore, #627610).

### Immunofluorescence Microscopy

Cells, grown on coverslips, were fixed with ice-cold methanol for 5 min, and rehydrated with PBS. Coverslips were incubated with 10% FBS (fetal bovine serum) for 1 h, and washed with PBS and then re-incubated with primary antibodies in 3% BSA (Sigma, #05470) for 1 h. Following three washes with PBS, the coverslips were further incubated in 1∶500 dilution of 2 mg/ml Alexa-488/Alexa-555/Alexa-647 (Molecular Probes) conjugated secondary antibodies, which were diluted in 3% BSA plus 5 µg/ml Hoechst 33342 (Molecular Probes), for 30 min. The coverslips were mounted in Prolong Gold Antifade (#P36930, Molecular Probes).

For Espl1-GFP localization, HeLa cells were pre-extracted in 0.1% TX-100 plus 20 µg/ml Alexa-647 conjugated nanobody (Chromotek) for 6 min, following 3 washes with PBS, the cells were directly observed in PBS without fixation.

For immunofluorescence analysis of separase in U2OS cells and co-localization of separase and centrosomal markers in HeLa Espl1-GFP cells, cells were pre-extracted with 0.1% TX-100 for either 1 min or 2 min, respectively, before fixation in ice-cold methanol for 5 min. Subsequent procedures were as above.

### Image Quantifications

The images were quantified using Fiji (ImageJ, http://fiji.sc/Fiji). The onset of anaphase was marked by the separation of sister chromatids. The mean fluorescence intensities of GFP and mCherry at the centrosomes were quantified using raw data without projection since the Scc1-based sensor, which also localizes to the nucleus, disperses to the cytoplasm after nuclear envelope breakdown to create a background signal. This makes quantifications of Z-projections of the sensor incomparable. The centrosome signals are usually observed in only 1 stack, as the distance between each stack was 1 µm (double the size of a centrosome). For the kendrin-based sensor, quantifications were made with Z-projected images as the sensor specifically localizes to the centrosome. For background correction, the cytoplasmic signal was subtracted from the measured centrosomal signal. The mCherry/eGFP (R_R_) ratio was used to represent sensor cleavage efficiency. In order to assess the rate of cleavage, each R_R_ value was normalized to the average of two measured R_R_ values. If the ratio was negative, it was considered to be zero. The graphs were plotted using Prism 6 software. For the H2B sensor, eGFP/mCherry ratio was used as the sensor was put C-terminally. For each quantification, n represents the number of centrosomes quantified.

The centriole distance in 3D has been measured using Fiji.

### Live Cell Imaging

Stable HeLa FRT cells were seeded onto Labtek Chambers (Thermo Scientific, #155411) and induced with 2 µg/ml doxycyclin (Sigma, #D9891) for 24 h before being observed in Live Cell Imaging Media (Gibco) using DeltaVision Olympus IX71 microscope (Applied Precision) equipped with DAPI, FITC, TRITC, and Cy5 filters (Chroma Technology) and CoolSNAP HQ camera (Photometrics). Images were taken every 6 min with 14 z-stack (1 µm/stack) using Plan Apo 60× NA 1.4 oil-immersion objectives (Olympus) with 2×2 binning.

### Metaphase Spread Experiment

Cells were grown in 6 cm dishes. 24 or 48 h after siRNA transfection, 100 ng/ml colcemid was added before a further incubation for 1 h (Kryomax, Invitrogen). The experiment was continued as before with a slight modification [57]. In brief, cells were collected and suspended in 0.8% sodium citrate (Sigma, #W302600), and incubated for 10 min. After sedimentation at 1,000 rpm for 10 min, cells were fixed with freshly prepared fixation solution (75% methanol+25% acetic acid) and incubated for 10 min. This process was repeated 3 times before final resuspending the cells in 300 µl of fixation solution and dropping them onto Fisher Superfrost/Plus slides (Thermo Scientific) followed by drying. Finally, the slides were incubated in 1 µg/µl Hoechst solution for 15 min and mounted with number 1.5 coverslips using Prolong Gold (Molecular Probes).

### Western Blot Sample Preparation

Cells were collected by scraping and washed with PBS. After lysis in 10 mM Tris-Cl pH 7.5; 150 mM NaCl; 5 mM EDTA; 0.1% SDS; 1% Triton X-100; 1% deoxycholate supplemented with 1 mM PMSF (Sigma) and Roche protease inhibitor cocktail for 30 min cell pellets were boiled with Laemmni buffer.

## Supporting Information

Figure S1 Design of separase sensors. (A) The scheme of the mCherry-Scc1^142-467^-eGFP construct. Two nuclear localization signals (NLS) were detected by cNLS Mapper. (B) The original mCherry-Scc1^(142-467)^-eGFP-PACT construct does not localize to centrosomes. 4-fold enlargements of boxes are depicted. Scale bar = 10 µm. (C) mCherry-Scc1^(142-467)-ΔNLS^-eGFP-PACT was only targeted to the centrosome when NLS-1 (319–323 aa of Scc1) was mutated (K^319^RKRK^323^ to N^319^GNGN^323^). It then colocalized with γ–tubulin and pericentrin. mCherry-Kendrin^(2059-2398)^-eGFP-PACT was targeted to the centrosome and colocalizes with both γ–tubulin and pericentrin. The inset on the right shows a two fold magnification of the boxed area in the main image. Scale bar = 10 µm. (D) FRAP analysis of mCherry-Scc1^(142-467)-ΔNLS^-eGFP-PACT at both the centrosome and within the nucleus. The mCherry-Scc1^(142-467)-ΔNLS^-eGFP-PACT construct stably associated with centrosomes. However, the nuclear pool was very mobile. n = 10. (E) The kendrin sensor was cleaved slightly before the metaphase-to-anaphase transition (upper panel and Figure 1). However, non-cleavable kendrin sensor (kendrin^NC^) was not cleaved (lower panel). The inset on the right shows a four fold magnification of the boxed area in the main image. Scale bar = 10 µm. (F) The fluorescent signal arising from the sensor at centrosomes in (E) was quantified. The bar represents SD, n>10.
(TIF)

Figure S2 Cleavage of sensors depends on separase (Espl1) activity. (A) Efficiency of RNAi mediated separase depletion in HeLa cells. Immunoblot analysis of mCherry-Scc1^(142-467)-ΔNLS^-eGFP-PACT and mCherry-Kendrin^(2059-2398)^-eGFP-PACT stably expressing HeLa cells upon Espl1 siRNA. An unspecific siRNA (UNS-i) was used as control. (B) Representative images of Scc1-sensor expressing HeLa cells after Espl1 siRNA treatment. The white arrows indicate the position of the unseparated chromosomes during cell division. The 4 fold enlargements show the centrosomes. The scale bar represents 10 µm. (C) Quantification of B. The cleavage of the sensor decreased upon siRNA depletion of Espl1. The panel shows the anaphase mCherry/eGFP ratio normalized to metaphase mCherry/eGFP ratio. Bar graphs: SEM, n>10. (D) mCherry-Kendrin-eGFP-PACT stably expressing HeLa cells were transfected with Espl1 siRNA. The cells that show a cut phenotype were analyzed for the cleavage of the separase sensor. The white arrows indicate the position of the unseparated chromosomes during cell division. The 4 fold enlargements show the centrosomes. The scale bar represents 10 µm. (E) Quantification of D. Performed as in (C).
(TIF)

Figure S3 H2B separase sensor cell line. (A) Structure of the chromosomal Scc1 sensor. (B) HeLa cells stably expressing eGFP-Scc1^(142-467)^-mCherry-H2B were analyzed every 6 min for the cleavage of the separase sensor on chromosomes. Separase activity on chromosomes was detected ∼6 min before anaphase onset (see Figure 1) [21]. Scale bar: 10 µm.
(TIF)

Figure S4 Cleavage of Scc1-based separase sensor during mitosis does not depend on caspase activity. (A) The cleavage of PARP as an indicator for caspase activity. Cells were arrest in prometaphase by STLC. Nocodazole, BI2536 or Ciliobrevin D were added after 24 h. Cells were then driven into G1 phase with the Cdk1 inhibitor RO-3336. PARP cleavage as judged by immunoblotting was used as an indication for caspase activation with UV treated cells serving as a positive control. (B) The experiment in Fig. 2B was repeated with a version of the Scc1-separase sensor that lacked the separase cleavage site (Scc1^NC^). No cleavage of the sensor was observed. 4-fold enlargements of the boxes are depicted. The scale bar represents 10 µm. (C) Experiment as Fig. 2B with wild-type Scc1-separase sensor in the presence of the apoptosis inhibitor Z-VAD. 4-fold enlargements of the boxes are depicted. The scale bar represents 10 µm.
(TIF)

Figure S5 Separase is recruited to centrosomes during mitosis. Espl1-eGFP expressing HeLa BAC cells were co-stained with γ–tubulin or pericentrin antibodies. The GFP signal was observed using 647 nm-conjugated nanobodies (upper panel). The signal at the centrosome and chromosome was enhanced when the cells were incubated in 0.1% Triton-X100 in PBS plus 20 µg/ml Alexa-647 conjugated Nanobody (Chromotek) for 6 min, following 3 washes with PBS. Cells were directly observed without fixation (lower panel). The arrows highlight the spindle poles. The scale bars represent 10 µm. Centrosomes are depicted as four-fold enlargement.
(TIF)

Figure S6 Centrosomal separase activity in response to Aki1, Sgo1 and astrin depletion. (A) Aki1 was depleted with 2 different siRNA oligonucleotides, Aki-1 and Aki-2. An unspecific UNS-i siRNA oligonucleotide was used as control. (B) Rootletin was absent from mitotic centrosomes after Sgo1/sSgo1, Aki1 or astrin depletion. Inlets of the right are four-fold enlargements of the boxes in the three color overlay. Scale bar represents 10 µm. (C) Sgo1/sSgo1 or astrin (Spag5) depletion does not cause premature centriole disengagement. Plk1 inhibition with BI2536 was used as negative control. The number of centrioles per spindle pole was used as a reference to determine the premature centriole disengagement. If there was only one centrin signal per pole, it was disengaged, if there were two centrin foci, they were engaged. (D) The distance between the mother and daughter centrioles marked by GFP-centrin2 was measured in 3D. STLC-arrested prometaphase cells were used as a negative control, whereas G1 cells were used as a positive control. G1 cells were chosen based on the chromatin structure as the cells were first arrested by STLC at prometaphase, and then driven out of mitosis into G1 via Cdk1 inhibition. 3 hours later, cells were fixed and analyzed. Statistical analysis was performed with one-way ANOVA (*** represents p<0.001, n = 30). (E) H2B-mCherry-Scc1^(142-467)^-GFP stably expressing HeLa cells were transfected with siRNAs to deplete either astrin (Spag5) or Sgo1 (targeting both sSgo1 and Sgo1) to determine whether separase was prematurely activated. In both cases, cells arrested in metaphase. Astrin depleted cells formed multipolar spindles without activating separase. The scale bar represents 10 µm. (F) HeLa cells were treated with either UNS or sSgo1 siRNA. Cells were fixed an analyzed by IF with the indicated antibodies. DNA was stained with DAPI. 4-fold enlargements were depicted. The arrows indicate γ-tubulin signals that do not colocalize with centrin. Scale bar: 10 µm.
(TIF)

Figure S7 Plk1 is important for the cleavage of the Scc1^(142-467)^-based separase sensor at chromosomes. (A) Scheme presentation of the Plk1 inhibition experiments. (B) H2B-mCherry-Scc1^(142-467)^-eGFP stably expressing HeLa cells were arrested in prometaphase with the Eg5 inhibitor STLC. Cells were incubated with or without the Plk1 inhibitor BI2536 followed by Cdk1 inhibition with RO-3336. Cells were analyzed every 6 min for the activity of separase on chromosomes upon Plk1 inhibition (BI2536). The scale bar represents 10 µm.
(TIF)

Figure S8 Plk1 regulates the activity of separase at substrate level throughout the cell. (A) mCherry to eGFP ratio quantifications of C, D, E; n = 20 cells. Error bars represent S.D. (B) mCherry-Scc1^(142-467)-**Δ**NLS-R172E^-eGFP-PACT sensor was partially cleaved; however, (C) the mCherry-Scc1^(142-467)-**Δ**NLS-R172E-S454A^-eGFP-PACT sensor was not cleaved. (D) The mCherry-Scc1^(142-467)-**Δ**NLS-S174A-S454A^-eGFP-PACT sensor was cleaved as the wild-type construct. (E) The mCherry-Scc1^(142-467)-**Δ**NLS-S174A-S454A^-eGFP-PACT stably expressing HeLa cells were arrested in prometaphase with STLC and then the Plk1 inhibitor BI2536 was added for 1 h. Cells were followed every 6 min upon Cdk1 inhibition to check the cleavage of the sensor. 3-fold enlargements of the centrosomes were depicted. (B–E) The scale bars represent 10 µm.
(TIF)

Figure S9 The cleavage of the first separase cut site (R172) of Scc1 at centrosomes also depends on Plk1. (A) The mCherry-Scc1^(142-467)-**Δ**NLS^-eGFP-PACT and The mCherry-Scc1^(142-467)-8A-**Δ**NLS-R450E/R460E^-eGFP-PACT cells were analyzed at metaphase and anaphase for sensor cleavage. 4-fold enlargements of the boxes are depicted (B) Quantification of (A). mCherry-Scc1^(142-467)-8A-**Δ**NLS-R450E/R460E^-eGFP-PACT sensor was only partially cleaved indicating that the first separase cleavage site in Scc1 is also activated at centrosomes by Plk1 phosphorylation. Bar graphs represents SEM, n = 20.
(TIF)
