# Supplementary figures and images for: Sensors at Centrosomes Reveal Determinants of Local Separase Activity
Source: PLoS Genet. 2014 Oct 9;10(10):e1004672. doi: 10.1371/journal.pgen.1004672 (PMC4191886; doi:10.1371/journal.pgen.1004672)

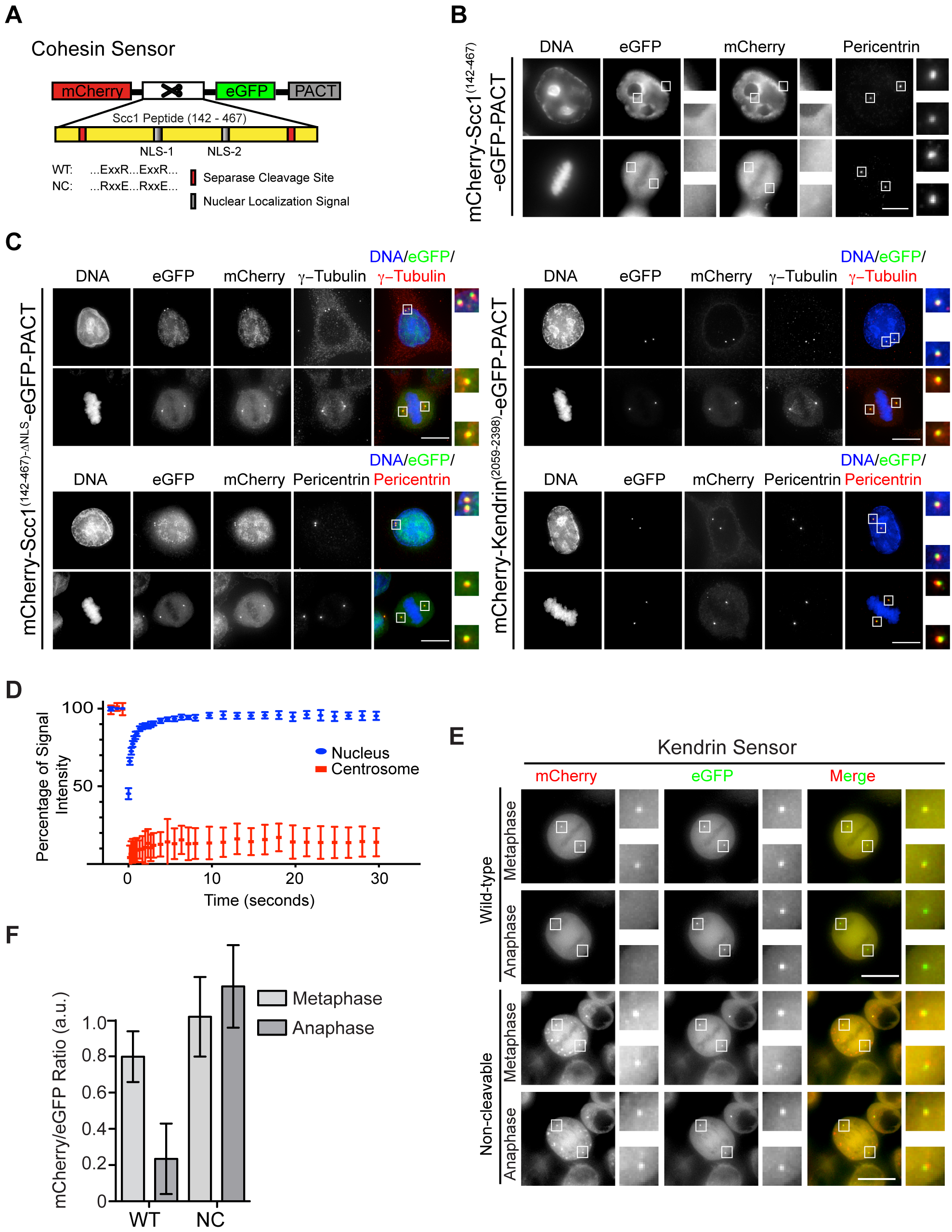

Supplement: Figure S1 — Design of separase sensors. (A) The scheme of the mCherry-Scc1142-467-eGFP construct. Two nuclear localization signals (NLS) were detected by cNLS Mapper. (B) The original mCherry-Scc1(142-467)-eGFP-PACT construct does not localize to centrosomes. 4-fold enlargements of boxes are depicted. Scale bar = 10 µm. (C) mCherry-Scc1(142-467)-ΔNLS-eGFP-PACT was only targeted to the centrosome when NLS-1 (319–323 aa of Scc1) was mutated (K319RKRK323 to N319GNGN323). It then colocalized with γ–tubulin and pericentrin. mCherry-Kendrin(2059-2398)-eGFP-PACT was targeted to the centrosome and colocalizes with both γ–tubulin and pericentrin. The inset on the right shows a two fold magnification of the boxed area in the main image. Scale bar = 10 µm. (D) FRAP analysis of mCherry-Scc1(142-467)-ΔNLS-eGFP-PACT at both the centrosome and within the nucleus. The mCherry-Scc1(142-467)-ΔNLS-eGFP-PACT construct stably associated with centrosomes. However, the nuclear pool was very mobile. n = 10. (E) The kendrin sensor was cleaved slightly before the metaphase-to-anaphase transition (upper panel and Figure 1). However, non-cleavable kendrin sensor (kendrinNC) was not cleaved (lower panel). The inset on the right shows a four fold magnification of the boxed area in the main image. Scale bar = 10 µm. (F) The fluorescent signal arising from the sensor at centrosomes in (E) was quantified. The bar represents SD, n>10. (TIF) [file pgen.1004672.s001.tif]

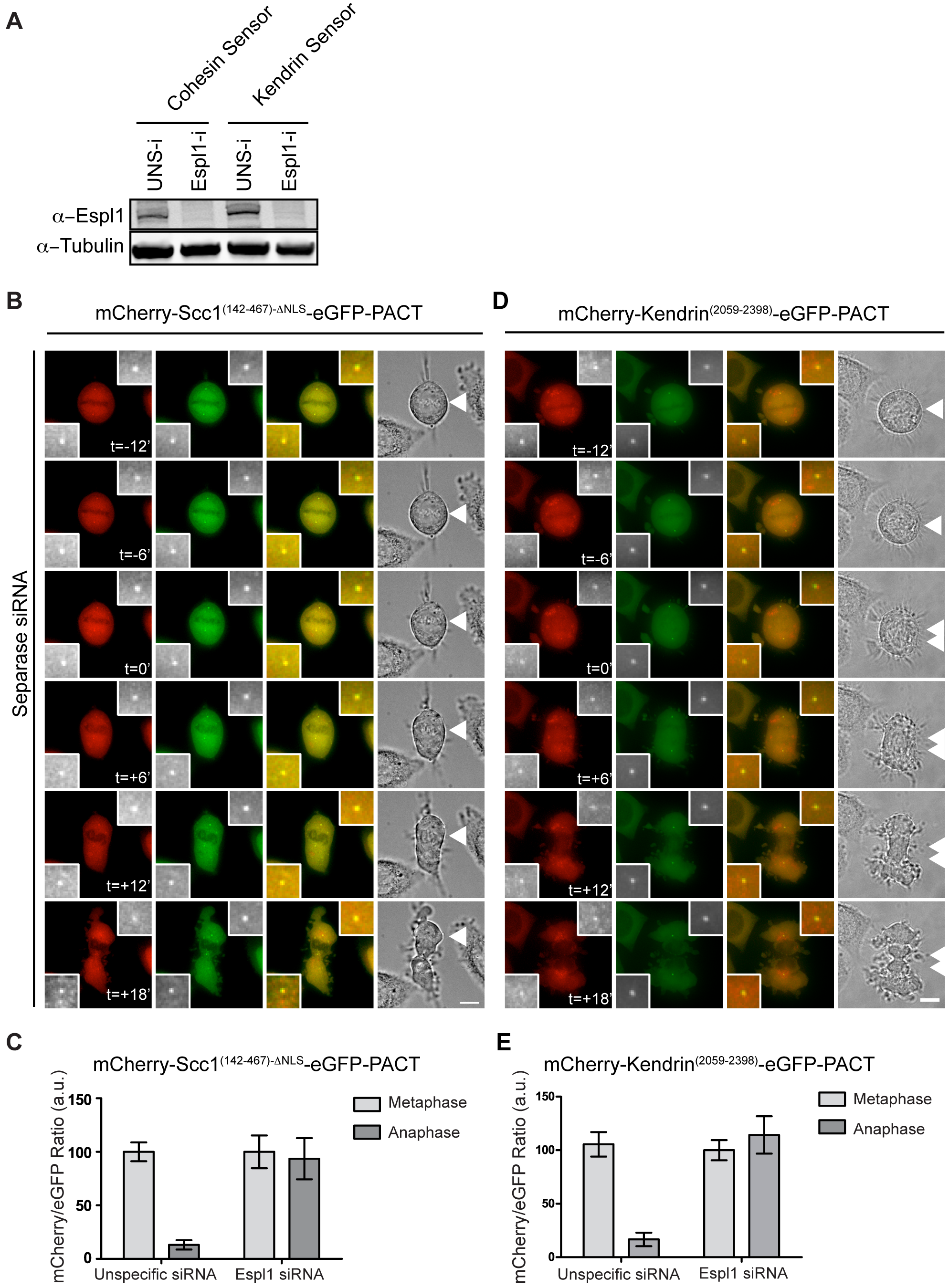

Supplement: Figure S2 — Cleavage of sensors depends on separase (Espl1) activity. (A) Efficiency of RNAi mediated separase depletion in HeLa cells. Immunoblot analysis of mCherry-Scc1(142-467)-ΔNLS-eGFP-PACT and mCherry-Kendrin(2059-2398)-eGFP-PACT stably expressing HeLa cells upon Espl1 siRNA. An unspecific siRNA (UNS-i) was used as control. (B) Representative images of Scc1-sensor expressing HeLa cells after Espl1 siRNA treatment. The white arrows indicate the position of the unseparated chromosomes during cell division. The 4 fold enlargements show the centrosomes. The scale bar represents 10 µm. (C) Quantification of B. The cleavage of the sensor decreased upon siRNA depletion of Espl1. The panel shows the anaphase mCherry/eGFP ratio normalized to metaphase mCherry/eGFP ratio. Bar graphs: SEM, n>10. (D) mCherry-Kendrin-eGFP-PACT stably expressing HeLa cells were transfected with Espl1 siRNA. The cells that show a cut phenotype were analyzed for the cleavage of the separase sensor. The white arrows indicate the position of the unseparated chromosomes during cell division. The 4 fold enlargements show the centrosomes. The scale bar represents 10 µm. (E) Quantification of D. Performed as in (C). (TIF) [file pgen.1004672.s002.tif]

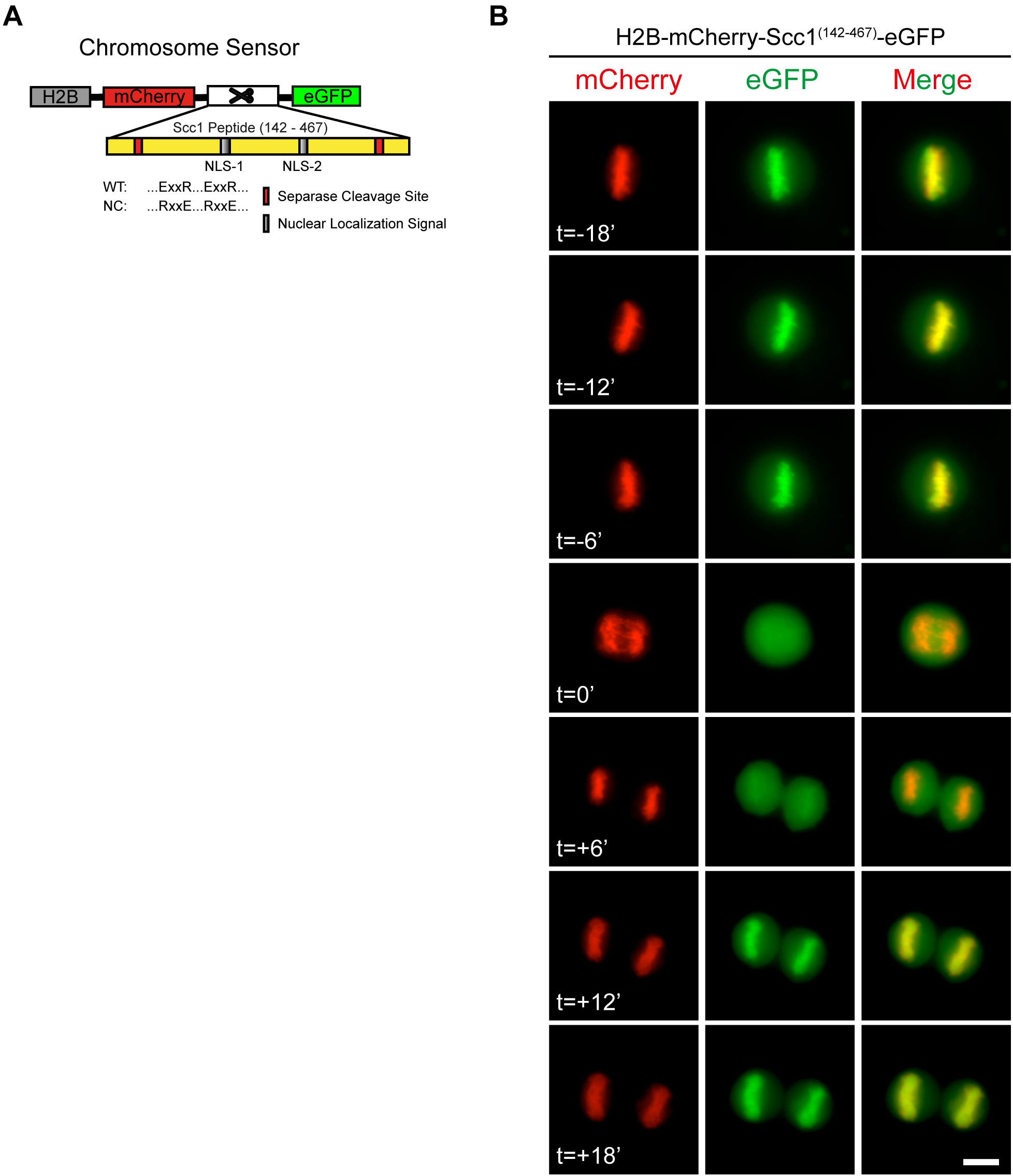

Supplement: Figure S3 — H2B separase sensor cell line. (A) Structure of the chromosomal Scc1 sensor. (B) HeLa cells stably expressing eGFP-Scc1(142-467)-mCherry-H2B were analyzed every 6 min for the cleavage of the separase sensor on chromosomes. Separase activity on chromosomes was detected ∼6 min before anaphase onset (see Figure 1) [21]. Scale bar: 10 µm. (TIF) [file pgen.1004672.s003.tif]

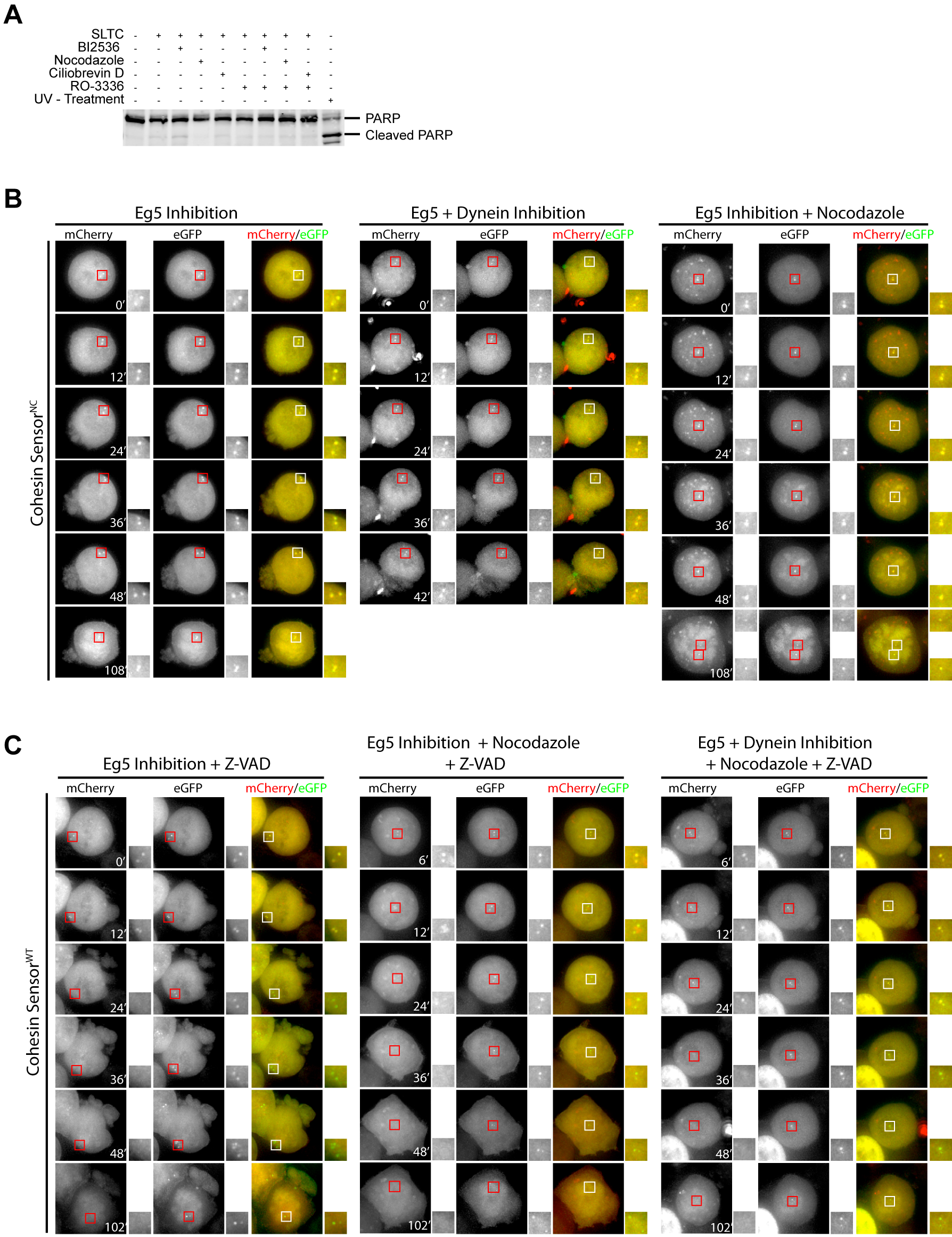

Supplement: Figure S4 — Cleavage of Scc1-based separase sensor during mitosis does not depend on caspase activity. (A) The cleavage of PARP as an indicator for caspase activity. Cells were arrest in prometaphase by STLC. Nocodazole, BI2536 or Ciliobrevin D were added after 24 h. Cells were then driven into G1 phase with the Cdk1 inhibitor RO-3336. PARP cleavage as judged by immunoblotting was used as an indication for caspase activation with UV treated cells serving as a positive control. (B) The experiment in Fig. 2B was repeated with a version of the Scc1-separase sensor that lacked the separase cleavage site (Scc1NC). No cleavage of the sensor was observed. 4-fold enlargements of the boxes are depicted. The scale bar represents 10 µm. (C) Experiment as Fig. 2B with wild-type Scc1-separase sensor in the presence of the apoptosis inhibitor Z-VAD. 4-fold enlargements of the boxes are depicted. The scale bar represents 10 µm. (TIF) [file pgen.1004672.s004.tif]

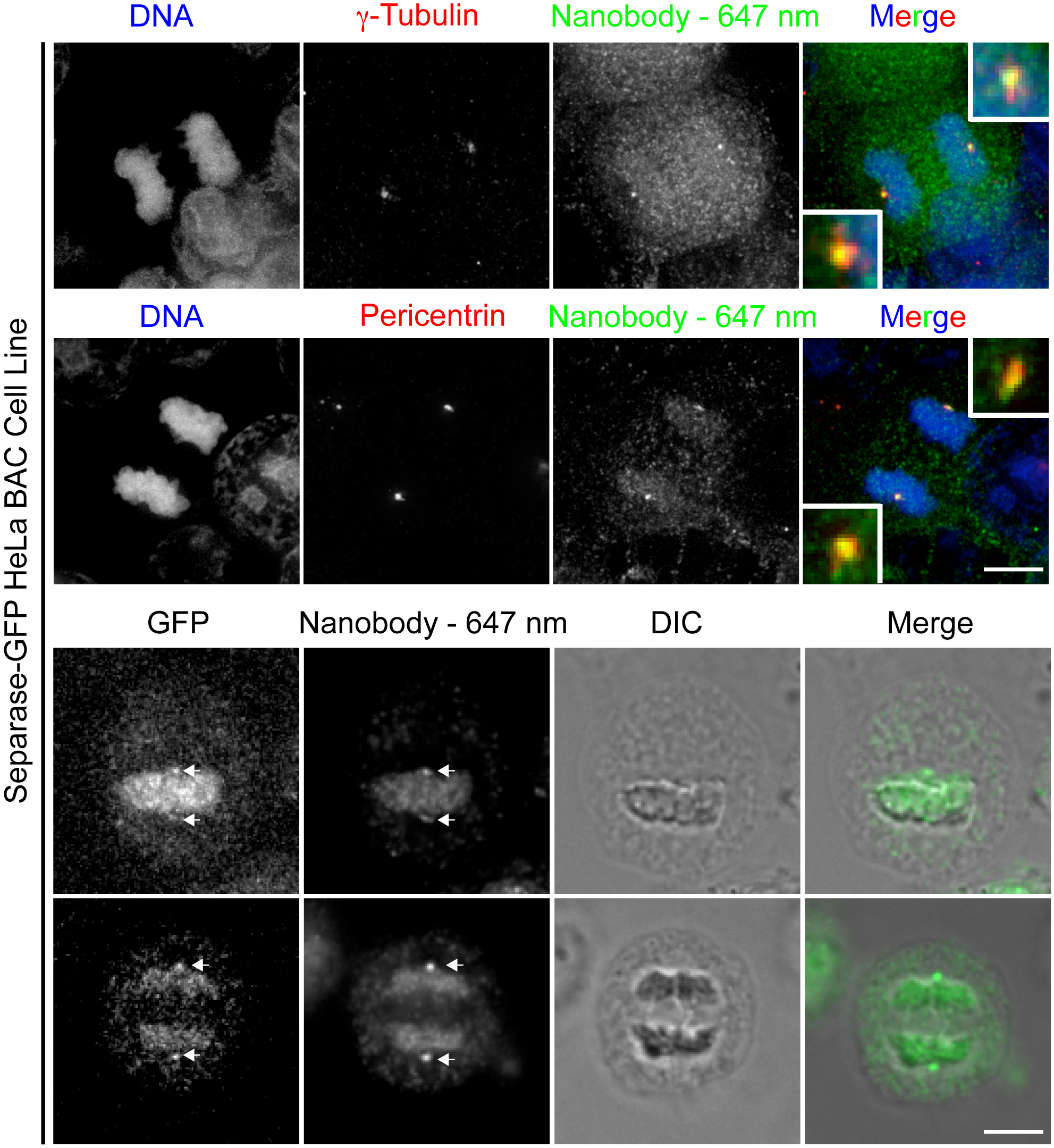

Supplement: Figure S5 — Separase is recruited to centrosomes during mitosis. Espl1-eGFP expressing HeLa BAC cells were co-stained with γ–tubulin or pericentrin antibodies. The GFP signal was observed using 647 nm-conjugated nanobodies (upper panel). The signal at the centrosome and chromosome was enhanced when the cells were incubated in 0.1% Triton-X100 in PBS plus 20 µg/ml Alexa-647 conjugated Nanobody (Chromotek) for 6 min, following 3 washes with PBS. Cells were directly observed without fixation (lower panel). The arrows highlight the spindle poles. The scale bars represent 10 µm. Centrosomes are depicted as four-fold enlargement. (TIF) [file pgen.1004672.s005.tif]

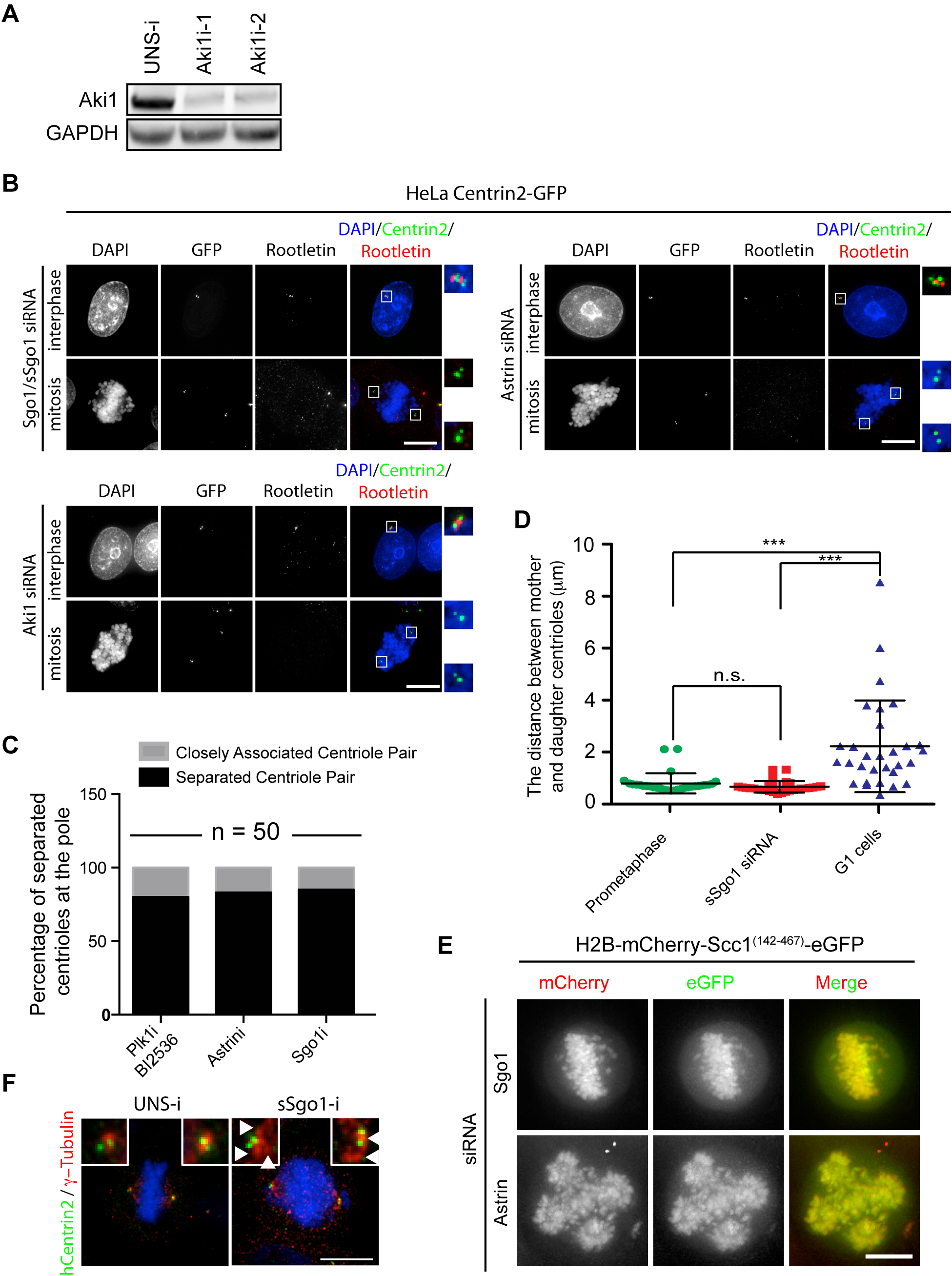

Supplement: Figure S6 — Centrosomal separase activity in response to Aki1, Sgo1 and astrin depletion. (A) Aki1 was depleted with 2 different siRNA oligonucleotides, Aki-1 and Aki-2. An unspecific UNS-i siRNA oligonucleotide was used as control. (B) Rootletin was absent from mitotic centrosomes after Sgo1/sSgo1, Aki1 or astrin depletion. Inlets of the right are four-fold enlargements of the boxes in the three color overlay. Scale bar represents 10 µm. (C) Sgo1/sSgo1 or astrin (Spag5) depletion does not cause premature centriole disengagement. Plk1 inhibition with BI2536 was used as negative control. The number of centrioles per spindle pole was used as a reference to determine the premature centriole disengagement. If there was only one centrin signal per pole, it was disengaged, if there were two centrin foci, they were engaged. (D) The distance between the mother and daughter centrioles marked by GFP-centrin2 was measured in 3D. STLC-arrested prometaphase cells were used as a negative control, whereas G1 cells were used as a positive control. G1 cells were chosen based on the chromatin structure as the cells were first arrested by STLC at prometaphase, and then driven out of mitosis into G1 via Cdk1 inhibition. 3 hours later, cells were fixed and analyzed. Statistical analysis was performed with one-way ANOVA (*** represents p<0.001, n = 30). (E) H2B-mCherry-Scc1(142-467)-GFP stably expressing HeLa cells were transfected with siRNAs to deplete either astrin (Spag5) or Sgo1 (targeting both sSgo1 and Sgo1) to determine whether separase was prematurely activated. In both cases, cells arrested in metaphase. Astrin depleted cells formed multipolar spindles without activating separase. The scale bar represents 10 µm. (F) HeLa cells were treated with either UNS or sSgo1 siRNA. Cells were fixed an analyzed by IF with the indicated antibodies. DNA was stained with DAPI. 4-fold enlargements were depicted. The arrows indicate γ-tubulin signals that do not colocalize with centrin. Scale bar: 10 µm. ( [file pgen.1004672.s006.tif]

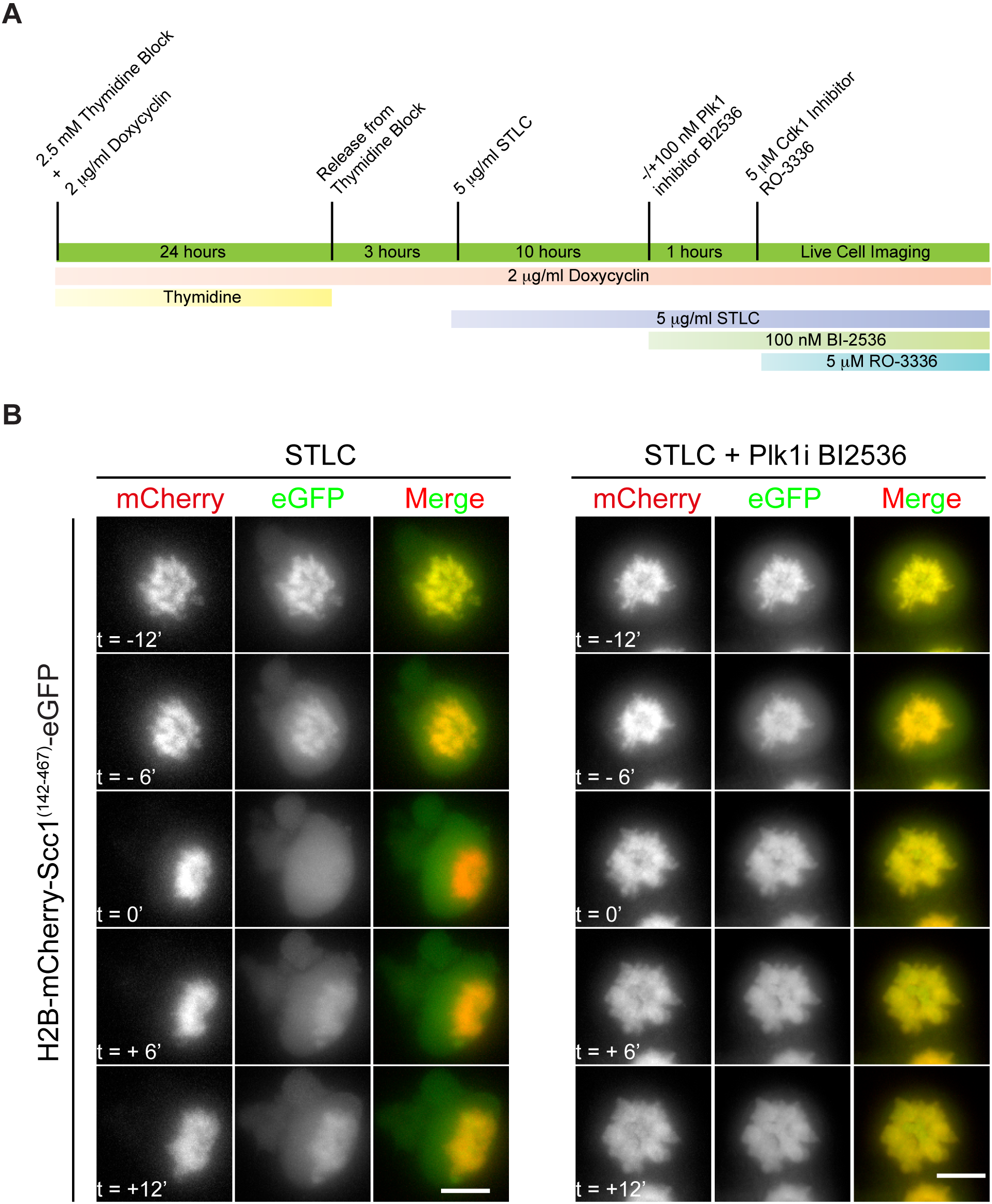

Supplement: Figure S7 — Plk1 is important for the cleavage of the Scc1(142-467)-based separase sensor at chromosomes. (A) Scheme presentation of the Plk1 inhibition experiments. (B) H2B-mCherry-Scc1(142-467)-eGFP stably expressing HeLa cells were arrested in prometaphase with the Eg5 inhibitor STLC. Cells were incubated with or without the Plk1 inhibitor BI2536 followed by Cdk1 inhibition with RO-3336. Cells were analyzed every 6 min for the activity of separase on chromosomes upon Plk1 inhibition (BI2536). The scale bar represents 10 µm. (TIF) [file pgen.1004672.s007.tif]

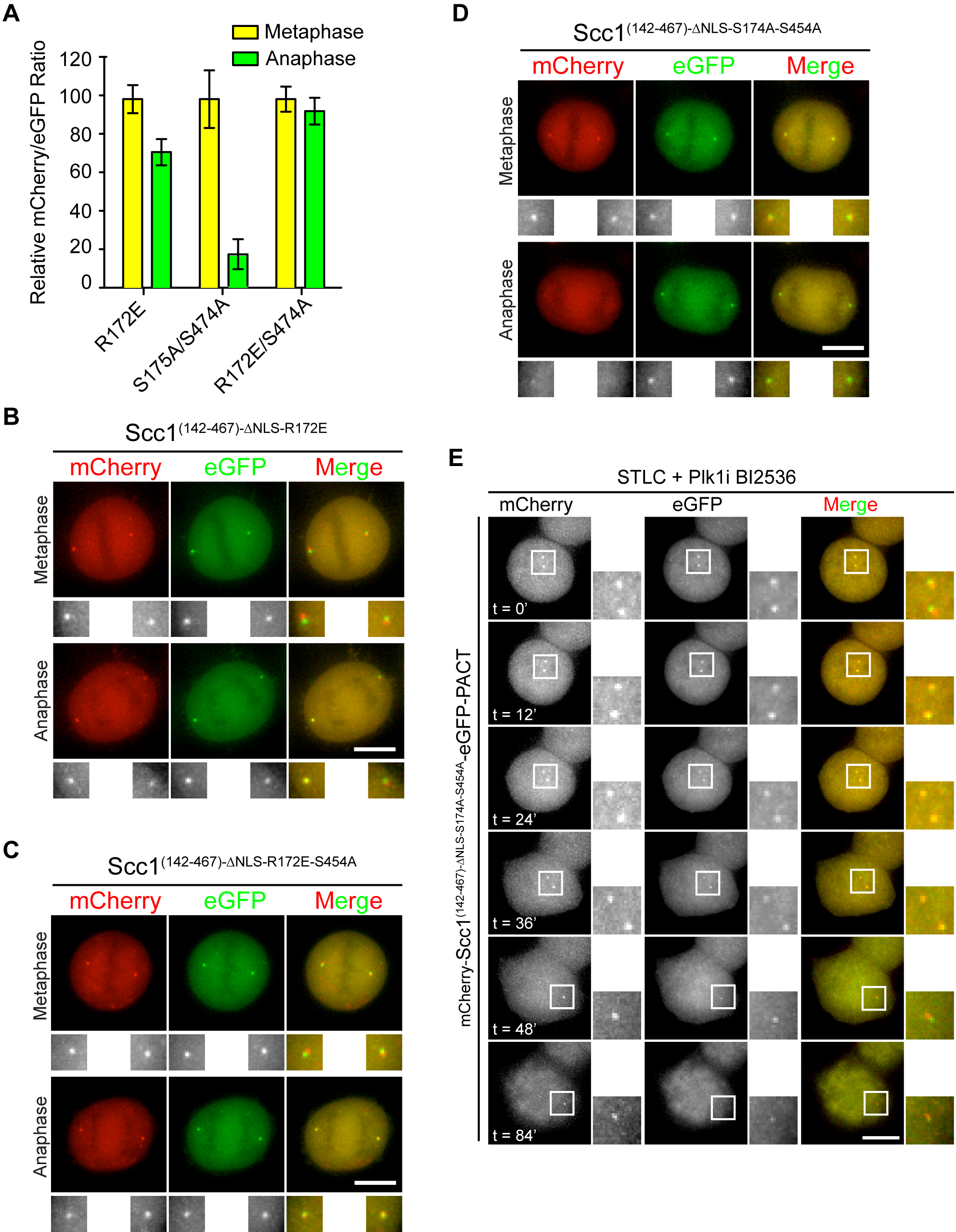

Supplement: Figure S8 — Plk1 regulates the activity of separase at substrate level throughout the cell. (A) mCherry to eGFP ratio quantifications of C, D, E; n = 20 cells. Error bars represent S.D. (B) mCherry-Scc1(142-467)-ΔNLS-R172E-eGFP-PACT sensor was partially cleaved; however, (C) the mCherry-Scc1(142-467)-ΔNLS-R172E-S454A-eGFP-PACT sensor was not cleaved. (D) The mCherry-Scc1(142-467)-ΔNLS-S174A-S454A-eGFP-PACT sensor was cleaved as the wild-type construct. (E) The mCherry-Scc1(142-467)-ΔNLS-S174A-S454A-eGFP-PACT stably expressing HeLa cells were arrested in prometaphase with STLC and then the Plk1 inhibitor BI2536 was added for 1 h. Cells were followed every 6 min upon Cdk1 inhibition to check the cleavage of the sensor. 3-fold enlargements of the centrosomes were depicted. (B–E) The scale bars represent 10 µm. (TIF) [file pgen.1004672.s008.tif]

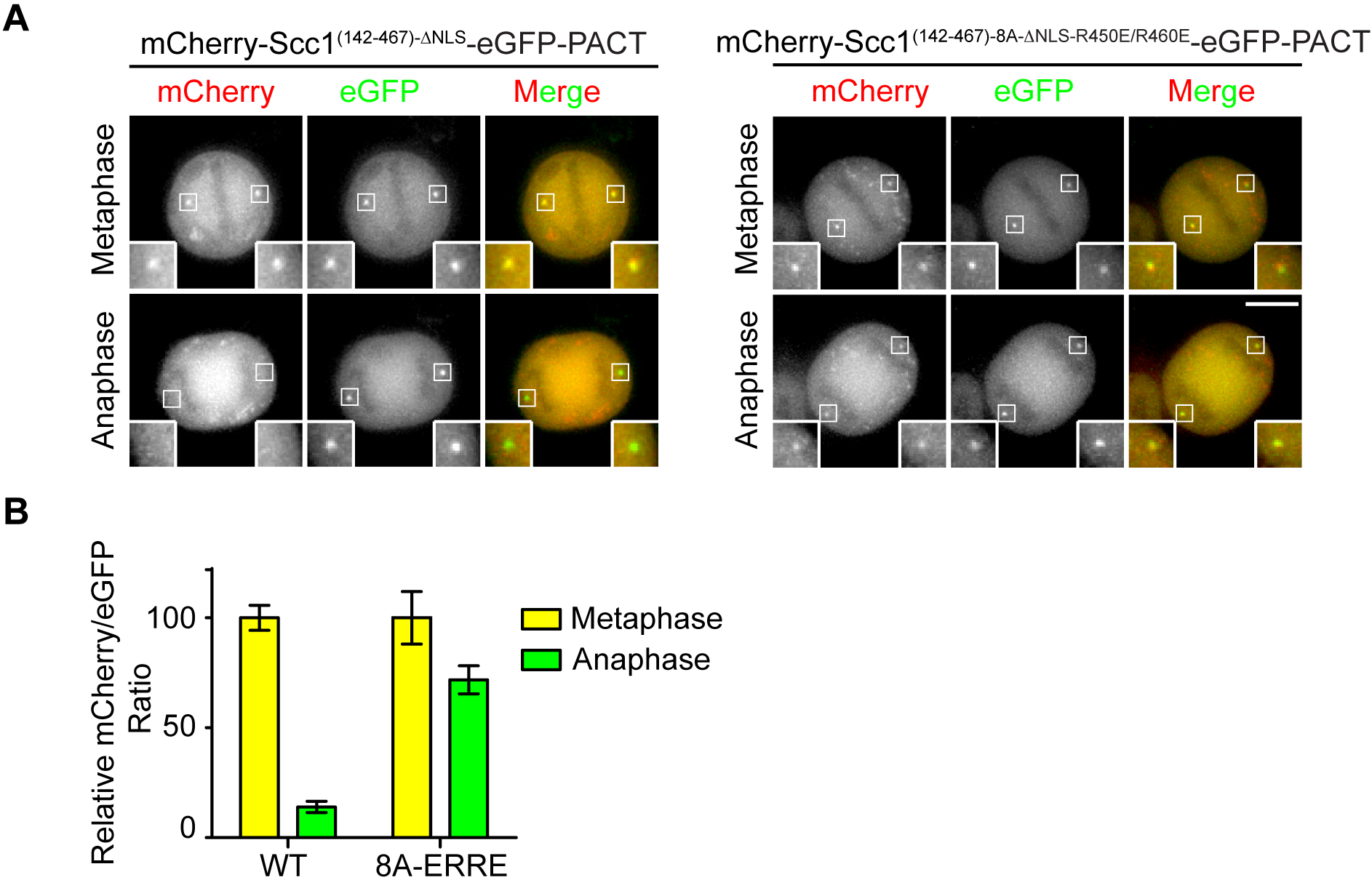

Supplement: Figure S9 — The cleavage of the first separase cut site (R172) of Scc1 at centrosomes also depends on Plk1. (A) The mCherry-Scc1(142-467)-ΔNLS-eGFP-PACT and The mCherry-Scc1(142-467)-8A-ΔNLS-R450E/R460E-eGFP-PACT cells were analyzed at metaphase and anaphase for sensor cleavage. 4-fold enlargements of the boxes are depicted (B) Quantification of (A). mCherry-Scc1(142-467)-8A-ΔNLS-R450E/R460E-eGFP-PACT sensor was only partially cleaved indicating that the first separase cleavage site in Scc1 is also activated at centrosomes by Plk1 phosphorylation. Bar graphs represents SEM, n = 20. (TIF) [file pgen.1004672.s009.tif]
